# Hypertonic treatment of acute respiratory distress syndrome

**DOI:** 10.3389/fbioe.2023.1250312

**Published:** 2023-10-23

**Authors:** Weiyu Li, Judith Martini, Marcos Intaglietta, Daniel M. Tartakovsky

**Affiliations:** ^1^ Department of Energy Science and Engineering, Stanford University, Stanford, CA, United States; ^2^ Department of Anaesthesia and Intensive Care Medicine, Medical University of Innsbruck, Innsbruck, Austria; ^3^ Department of Bioengineering, University of California, San Diego, San Diego, CA, United States

**Keywords:** ARDS, hypertonic treatment, intravascular infusion, pulmonary, mathematical model

## Abstract

Many viral infections, including the COVID-19 infection, are associated with the hindrance of blood oxygenation due to the accumulation of fluid, inflammatory cells, and cell debris in the lung alveoli. This condition is similar to Acute Respiratory Distress Syndrome (ARDS). Mechanical positive-pressure ventilation is often used to treat this condition, even though it might collapse pulmonary capillaries, trapping red blood cells and lowering the lung’s functional capillary density. We posit that the hyperosmotic-hyperoncotic infusion should be explored as a supportive treatment for ARDS. As a first step in verifying the feasibility of this ARDS treatment, we model the dynamics of alveolar fluid extraction by osmotic effects. These are induced by increasing blood plasma osmotic pressure in response to the increase of blood NaCl concentration. Our analysis of fluid drainage from a plasma-filled pulmonary alveolus, in response to the intravenous infusion of 100 ml of 1.28 molar NaCl solution, shows that alveoli empty of fluid in approximately 15 min. These modeling results are in accordance with available experimental and clinical data; no new data were collected. They are used to calculate the temporal change of blood oxygenation, as oxygen diffusion hindrance decreases upon absorption of the alveolar fluid into the pulmonary circulation. Our study suggests the extraordinary speed with which beneficial effects of the proposed ARDS treatment are obtained and highlight its practicality, cost-efficiency, and avoidance of side effects of mechanical origin.

## 1 Introduction

Acute respiratory distress syndrome (ARDS), caused by various infectious and non-infectious agents, is associated with the hindrance of blood oxygenation due to the accumulation of cells and fluid in the lung alveoli. This phenomenon is caused by cytokines activating immune cells. It leads to the accretion of fluid and cellular debris in the lungs, a condition manifesting itself in the clinical picture of ARDS (Tsonas et al., 2021). This pathology came to light during the Vietnam War, while treating hemorrhagic shock with massive infusions of Ringer’s lactate. Since the latter lacks oncotic properties, it caused an imbalance of the Starling-Landis-Pappenheimer (SLP) fluid exchange process (Pappenheimer and Soto-Rivera, 1948; Landis, 1963), favoring lung edema at the rate of one blood volume per day. Comparison of ARDS conditions arising from different origins indicates that their effect and treatment should be similar, particularly regarding the implementation and use of mechanical ventilation, with shared benefits and problems (Auld et al., 2020).

In a healthy organism, the balance of blood/tissue fluid exchange is due to the interaction of hydraulic, osmotic, and oncotic pressures that takes place in the microcirculation. This process, regulated by the control of capillary blood pressure and oncotic gradients, allows the transport and containment of blood in water-permeable blood capillaries. Direct *in vivo* measurements of fluid exchange and capillary hydraulic and osmotic/oncotic pressures (Intaglietta and Zweifach, 1974) provide a quantitative assessment of these physiologic phenomena. In combination with lymph-flow management, blood/tissue exchange collects tissue fluid and byproducts of tissue metabolism for detoxification in the venous system.

The lung presents an additional factor in regulating transcapillary fluid flow due to the air pressure acting at the fluid/atmosphere interface. Ventilators manipulate the latter by controlling the composition, pressure, and rate of delivery of the inspired air. In doing so, ventilation-related mechanical parameters are superposed onto those induced by the fluid hydraulic and oncotic pressure gradients of the SLP fluid exchange system. The mechanical effects of hydraulic and oncotic pressure (COP) interaction in the lung subjected to ventilation-augmented pressure manifest themselves only in the pulmonary microcirculation, since the rest of the organism remains subjected to the environmental pressure.

Mechanical ventilation, a frequently used treatment for pulmonary ventilatory insufficiency, allows one to control inspired air pressure, rate of delivery, and composition. Generally, mechanical ventilation increases air delivery pressure above atmospheric during the inspiration portion of the respiratory cycle. The pressure is lowered during expiration, but maintained at a lower, above atmospheric, positive end-expiratory pressure (PEEP) to prevent lung collapse. The ventilation pressure effect, Δ*P*_vent_, is defined as the average pressure in the inspiration/expiration cycle; it is an additional hydraulic pressure exerted on alveolar fluid, thus affecting hydraulic tissue pressure in the SLP system. Specifically, it affects both the rate of fluid extravasation from capillaries (Intaglietta and Zweifach, 1974) and the compression of pulmonary capillaries, potentially affecting the lung’s functional capillary density (FCD), the number of capillaries per unit volume of tissue with the passage of red blood cells (RBCs) (Kerger et al., 1996).

The literature provides a somewhat contradictory evaluation of the effectiveness of ventilation pressure (Auld et al., 2020; Tsonas et al., 2021), whose maximum peak value is between 5 and 14 cm H2O above ambient. Each case might be unique given many associated variables; however, the increase in average inspiration pressure tends to collapse pulmonary capillaries, trapping RBCs and lowering FCD. The critical issue is whether the increased alveolar gas pressure due to pressure ventilation can safely lower and ultimately eliminate the volume of the extra-capillary fluid, thus decreasing the diffusional resistance to gas exchange due to fluid accumulating in the pulmonary alveoli. Another consideration is the apparent significant incidence of lung injury associated with mechanical ventilation (Auld et al., 2020).

The limitations of this technique, reviewed in detail in Beitler et al. (2016), stem from the mechanical trauma caused to lung microcirculation when air pressure and capillary blood flow velocity exceed their limiting values. An analysis of the incidence of perioperative lung injury and its effect on postoperative mortality suggest that “… definitive evidence of its benefit for the general surgical population does not exist” (O’Gara and Talmor, 2018). A multicenter observational cohort study of 223 critically ill patients with COVID-19, found ICU mortality to be 35%, while mechanical ventilation, used in 75% of the cases, was associated with a mortality rate of 44% (Roedl et al., 2021). Furthermore, mechanical ventilators might be necessary for as long as a month to achieve the required palliative condition (Nolley et al., 2023), a prolonged period in which barotrauma damage might initiate and extend (Amado-Rodríguez et al., 2017). These factors suggest that an alternative should be contemplated.

We posit that the hyperosmotic-hyperoncotic infusion should be explored to treat pulmonary fluid accumulation-induced respiratory impediments, which has the potential added benefit of improving systemic hemodynamics and microvascular function. Our hypothesis is supported by the concept of “hypertonic shock resuscitation” (Velasco et al., 1980; Rocha-e Silva et al., 1986; Cabrales et al., 2009) for treating acute hemorrhagic shock. It arises from the observation that the intravenous introduction of hypertonic saline into the circulation of affected subjects, 1/10 of shed volume of 7.5% NaCl, led to the fast recovery of arterial blood pressure. This effect is mostly attributed to increased myocardial contractility, vascular constriction in different compartments, and the rapid restoration of blood volume during the period in which the blood-tissue NaCl concentration difference is maintained. Subsequent studies (Smith et al., 1985) found that adding a hyperoncotic component (6% dextran 70 kDa) to the infusion solution further improved outcomes, prolonging the duration of beneficial microvascular effects.

Hypertonic perfusion might be a supportive treatment in ARDS because it induces the rapid resorption of tissue fluid into the vascular compartment (Powers Jr et al., 1977; Mazzoni et al., 1988; Sobhy et al., 2020). The physiological explanation for this phenomenon stems from the experimental evidence (Mazzoni et al., 1989, 1990) that the endothelial cells are an additional osmotic compartment in the capillary-tissue fluid exchange process in the SLP system since their volume—regulated by the same hydraulic, osmotic, and oncotic forces—directly and beneficially increases capillary lumen, blood flow, and FCD. Moreover, experimental evidence (Berthiaume and Matthay, 2007) indicates that, by acting as a barrier to the movement of fluid into the alveoli, the alveolar surface actively modulates the transport of ions and solutes. This process promotes edema fluid clearance, thus supporting the treatment of ARDS by non-mechanical approaches.

As a first step in verifying the feasibility of the proposed ARDS treatment, we model the dynamics of alveolar fluid extraction by osmotic effects. These are induced by raising blood plasma osmotic pressure in response to the increase of blood NaCl concentration to 0.176, 0.197, and 0.218 molar. Model results are then used to calculate the temporal change of blood oxygenation, as O_2_ diffusion hindrance decreases upon absorption of the alveolar fluid into the pulmonary circulation.

## 2 Methods

We present a mathematical model of alveolar fluid extraction facilitated by increase of blood NaCl concentration and, consequently, of blood plasma osmotic pressure. Our model is distinct from, and complementary to, the extensive modeling work on the ARDS pathology (Cinkotai, 1974; Dong et al., 2022) and its ventilation-based treatment (Morton et al., 2019). These models focus on the effects of the extra fluid on gas flow in the lung, without addressing how the rapid elimination of this fluid by osmotic pressure facilitates oxygen access to the lung capillaries. Our study does not involve human subjects and animal research. Consequently, no IRB review was necessary (and thus no number was assigned) because it did not fall under the board’s guidelines as human subjects research.

**FIGURE 1:**
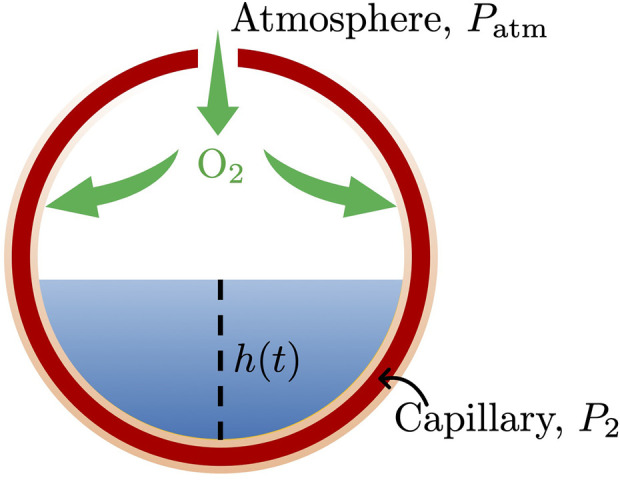
Oxygen exchange between an alveolus, partially filled with water with height *h*(*t*) and density *ρ*, and the surrounding capillary sheet-like envelope. The empty alveolar space is connected to the atmosphere, with pressure *P*_atm_, and separated from the surrounding capillary, with hydrostatic pressure *P*_2_, by a semi-permeable alveolar membrane, with hydraulic conductivity *K*.

Alveoli are modeled as hollow spheres with radius *R* surrounded by capillaries (Figure 1). The alveoli are connected to the open atmosphere with pressure *P*_atm_; a semi-permeable alveolar membrane, with hydraulic conductivity *K*, separates them from the surrounding capillaries with hydrostatic pressure *P*_2_. The affected alveoli are partially filled with water at a height *h*(*t*) and density *ρ*. Oxygen exchange $Q_{{\text{O}}_{2}}$ takes place between the capillaries and the alveolar surface above water. This physiological conceptualization is adapted from the modeling study (Sobin et al., 1970), which found pulmonary capillaries to be in such proximity as to justify the assumption of blood flowing between endothelial sheets held together by pillar-like connections occurring at the frequent pulmonary capillary bifurcations.

**FIGURE 2:**
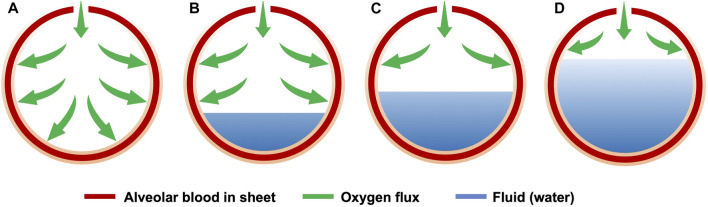
Schematic of oxygen exchange between a healthy alveolus **(A)** and affected alveoli that are 20% **(B)**, 50% **(C)**, or 80% **(D)** fluid filled.

In a healthy alveolus (Figure 2A), i.e., when *h*(*t*) = 0, the total oxygen delivery $Q_{{\text{O}}_{2}}$ to the capillaries is proportional to the total surface area *A* = 4*πR* ^2^,

$$Q_{{\text{O}}_{2}}\propto A.$$ (1)

Unhealthy alveoli (Figures 2B–D) are partially filled with water, whose height is *h*(*t*), and oxygen delivery, $Q_{{\text{O}}_{2}}^{*}$ , is proportional to the surface area above the water, *A* − A*,

$$Q_{{\text{O}}_{2}}^{*}\propto A-A^{*},$$ (2)

where $A^{*}=2\pi Rh\left(t\right)$ . We use Starling’s equation (Intaglietta and Zweifach, 1974),

$$Q_{\text{w}}=KA^{*}\left[P_{1}-P_{2}-\sigma \left(\pi_{1}-\pi_{2}\right)\right],$$ (3)

to describe the net flow of water *Q*_w_ between the alveoli and the capillary sheet across the semi-permeable alveolar membrane. Here, *K* is the hydraulic conductivity of the membrane (m/s/atm); *σ* is the Staverman’s reflection coefficient, a unitless constant characterizing the membrane’s permeability to a given solute. The parameters *P*_1_, *P*_2_, *π*_1_ and *π*_2_ are hydrostatic pressure and colloid osmotic pressure (oncotic pressure) in the interstitial fluid (water) and capillary, respectively. Given the small size of the alveoli, the hydrostatic pressure in the water, *P*_1_, is approximately equal to the atmospheric pressure, *P*_atm_, at any point below the water table. The net water flow is defined as

$$Q_{\text{w}}=v_{1}A_{1}.$$ (4)

where $A_{1}=\pi \left[R^{2}-{\left(R-h\left(t\right)\right)}^{2}\right]$ is the surface area of the fluid whose free surface height is *h*(*t*), and the flow velocity *v*_1_ at the free surface $x=h\left(t\right)$ is given by the rate of change of the height according to

$$v_{1}=-\frac{\text{d}h}{\text{d}t}.$$ (5)

We assume that, in the absence of treatment, there is a force balance before saline injection $P_{1}-P_{2}-\sigma \left(\pi_{1}-\pi_{2}\right)=0$ such that *Q*_w_ = 0.

After introducing hypertonic saline, the net flow of fluid is driven by the osmotic pressure’s change in the capillary, Δ*π*_o_. Combining Eqs 3–5, we obtain

$$-\frac{\text{d}h}{\text{d}t}\pi \left[R^{2}-{\left(R-h\right)}^{2}\right]=-2\pi KRh\sigma \Delta \pi_{\text{o}},$$ (6)

where *π*_o_ is the osmotic pressure in the capillary after the injection of *n* units (*n* × 100 mL) of hypertonic saline (7.5% w/v). According to the van’t Hoff theory (Van’t Hoff, 1888)

$$\pi_{\text{o}}=iCR_{\text{c}}T,$$ (7)

where *i* is the dimensionless van’t Hoff index, *R*_c_ is the ideal gas constant (L⋅atm/K/mol), and *T* is the absolute temperature (K). The molar concentration, *C*, of NaCl in the capillary after the injection of *n* units (*n* × 100 mL) of hypertonic saline (7.5% w/v) into 5 L of blood is related to the NaCl concentration, *C*_b_, in the blood before the injection of hypertonic saline by (Kreimeier and Messmer, 1992)

$$C=\left(C_{\text{h}}\times n\times 0.1+C_{\text{b}}\times 5\right)/\left(5+n\times 0.1\right)\;\text{mol/L},$$ (8)

where *C*_h_ is NaCl concentration of hypertonic saline. The change of the osmotic pressure in the capillary, Δ*π*_o_ is

$$\Delta \pi_{\text{o}}=i\left(C-C_{\text{b}}\right)\;R_{\text{c}}T,$$ (9)

Integrating Eq. 6 yields an expression for the water height in the alveolus,

$$h\left(t\right)=2R-\sqrt{{\left(2R-h_{0}\right)}^{2}+4KR\sigma \Delta \pi_{\text{ot}}}.$$ (10)

This expression implies that the total time, *t*_total_, it takes to drain the alveoli, $h\left(t_{\text{total}}\right)=0$ , is

$$t_{\text{total}}=\frac{4Rh_{0}-h_{0}^{2}}{4KR\sigma \Delta \pi_{\text{o}}}.$$ (11)

The corresponding prediction of temporal change in the normalized oxygen delivery $Q_{{\text{O}}_{2}}^{*}/Q_{{\text{O}}_{2}}$ is

$$\frac{Q_{{\text{O}}_{2}}^{*}}{Q_{{\text{O}}_{2}}}=\frac{A-A^{*}}{A}=\frac{2R-h\left(t\right)}{2R}=\frac{\sqrt{{\left(2R-h_{0}\right)}^{2}+4KR\sigma \Delta \pi_{\text{o}}t}}{2R}.$$ (12)

Like any mathematical model, ours provides a simplified description of reality. Transport of O_2_ from the gas phase into blood is impeded by water interposed between the gas source and blood; in normal ambient conditions, O_2_ solubility in air is approximately 30 times higher than in water (Li et al., 2021), and the diffusion constant of O_2_ is 10,800 times greater in air than in water. These major disparities suggest that maximal transferal of O_2_ from atmosphere to blood is achieved by minimizing water/gas alveolar volume ratios. In describing this phenomenon, our model assumes that the air and water phases are separated by a plane. Given the small size of alveoli, surface tension changes may introduce some variability in the shape of the gas/fluid interface leading to volume configurations that favor gas transfer in the “wetter” alveoli. Additional complications stem from the alveolar gas entry position relative to the gravitational direction; this variable, related to patient posture, diminishes periods in which alveolar gas inlets become fully occluded by gravity acting on the accumulated alveolar fluid. These effects tend to be small in the normal lung due to the presence of pulmonary surfactants that lower water surface tension by a factor of three (Gregory et al., 1991). Many lung ailments, including COVID-19 infection, are accompanied by a physiologically significant decrease in surfactant production. The introduction of lung surfactants in COVID-19 patients has been proposed as a pharmacological intervention (Busani et al., 2020; Wang et al., 2021).

## 3 Results

**FIGURE 3:**
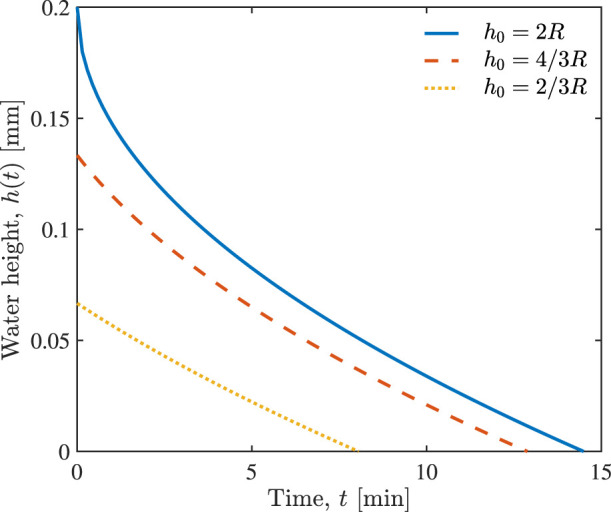
Temporal evolution of water height *h*(*t*) in the alveolus in response to the injection of *n* = 1 unit of hypertonic saline, for the alveoli that are fully, 2/3 and 1/3 filled with water which have different initial water heights *h*_0_.

Values of the parameters used in our simulation are presented in Table 1; they come from the literature, and none of them were used for data fitting. Figure 3 shows the temporal evolution of the water height *h*(*t*) in an alveolus, predicted by Eq. 10, for different initial water heights *h*_0_. It represents the drainage of the alveolus (fully, 2/3 or 1/3 filled with water) caused by the injection of *n* = 1 unit of hypertonic saline. It takes approximately 8, 13, or 15 min to drain alveoli that are respectively 1/3, 2/3 or fully water filled. Figure 3 also shows that the drainage velocity *v*_1_ at the free surface, *x* = *h*(*t*), decreases with time *t*.

**TABLE 1:** Parameters used in the simulations.

Parameter	Symbol	Value	Units	Reference
Radius of alveoli	*R*	0.1	mm	Ochs et al. (2004)
Atmospheric pressure	*P*_atm_	1	atm	
NaCl concentration in blood (0.9% w/v)	*C*_b_	0.154	mol/L	
NaCl concentration of hypertonic saline (7.5% w/v)	*C*_h_	1.28	mol/L	Kreimeier and Messmer (1992)
Van’t Hoff index of NaCl	*i*	2	—	
Ideal gas constant	*R*_*c*_	0.0821	L⋅atm/K/mol	
Temperature	*T*	310	K	
Hydraulic conductivity of the membrane	*K*	3.4 ⋅ 10^–7^	m/s/atm	Renkin (1977)
Reflection coefficient	*σ*	0.3	—	Perl et al. (1975)

**FIGURE 4:**
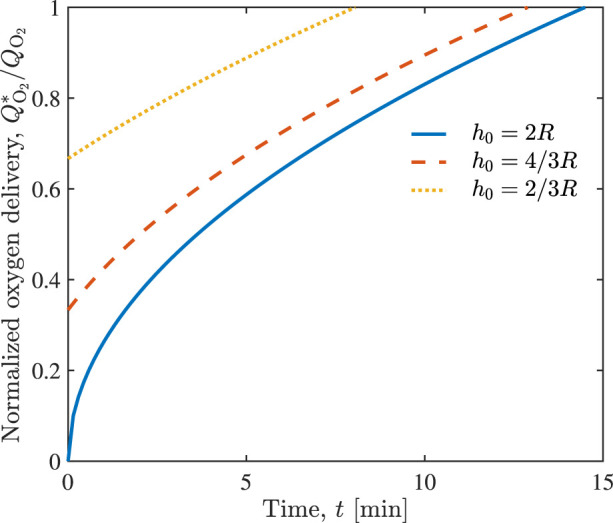
Temporal evolution of normalized oxygen delivery $Q_{{\text{O}}_{2}}^{*}/Q_{{\text{O}}_{2}}$ in the alveolus in response to the injection of *n* = 1 unit of hypertonic saline, for the alveoli that are fully, 2/3, and 1/3 water filled, having respectively different initial water heights *h*_0_.

The corresponding improvement in normalized oxygen delivery $Q_{{\text{O}}_{2}}^{*}/Q_{{\text{O}}_{2}}$ , predicted by Eq. 12, is shown in Figure 4. The injection of *n* = 1 unit of hypertonic saline restores oxygen delivery to its normal level in approximately 8, 13, and 15 min, depending on whether the alveolus was initially 1/3, 2/3, or fully water filled. These times are the same as those necessary to drain the alveoli (Figure 3).

**FIGURE 5:**
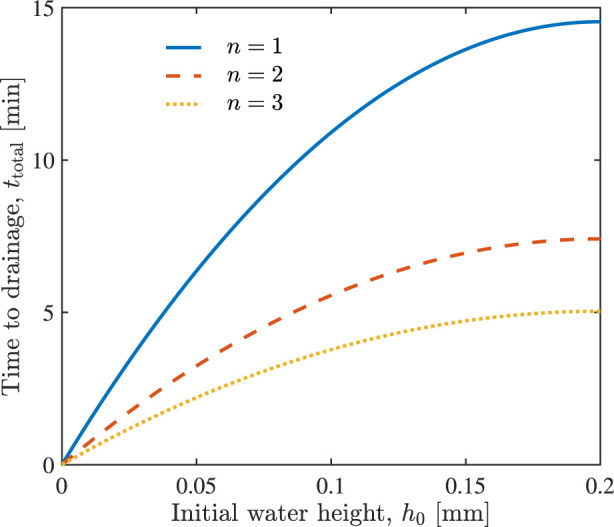
The total time required to drain variously filled alveoli (various initial water height *h*_0_) through the injection of *n* = 1, 2 and 3 units of hypertonic saline.

Figure 5 shows the time, *t*_total_, necessary to drain alveoli, with initial water height *h*_0_, when *n* = 1, 2 and 3 units of hypertonic saline are injected, according to Eq. 11. The injection of more hypertonic saline accelerates water drainage from the alveoli. For example, it takes around 15, 7, and 5 min to drain the alveoli filled with *h*_0_ = 0.2 mm of water, if *n* = 1, 2, and 3 units of hypertonic saline were injected, respectively.

## 4 Discussion

Our analysis of fluid drainage from a plasma-filled pulmonary alveolus, in response to the intravenous infusion of 100 ml of 1.28 molar NaCl solution, shows that alveoli empty of fluid in approximately 15 min. We posit that this effect could constitute a therapeutic approach for treating edema in general and particularly lung edema, caused by heart failure or fluid overload and consecutive lung edema due to acute kidney failure. This approach would appear to be directly applicable in these syndromes presenting relatively uniform pathophysiological conditions, void of associated or causative inflammatory stimuli.

Current clinical understanding of the problems arising from lung edema indicates that PEEP ventilation is an effective treatment, since it “pushes” the fluid out of the alveoli and back into the tissue to be eventually reabsorbed by venous capillaries and lymphatic vessels. It is an established fact that in acute emergency conditions, when oxygen must be supplied within minutes, mechanical ventilation is a critical, unique, and necessary intervention.

Mechanical ventilators, however, affect microvascular function when used to support lung ventilation; they can negatively influence the lung ventilation’s effectiveness, significantly prolonging a ventilator’s usage, ultimately negating its positive impact seen in acute interventions. This is a consequence of the mechanical forces impacting lung microcirculation and particularly the lung capillary walls, which are subjected to an increase of extravascular pressure in the presence of constant intravascular pressure. This balance between intra and extravascular pressures lowers the net capillary distending intravascular pressure, decreases capillary cross-section, and significantly affects capillary blood flow, which is dependent on the 4th power of the capillary radius. Thus, mechanical ventilation could present a handicap in terms of ensuring adequate capillary blood flow necessary to ensure a sufficient supply of oxygenated blood.

This problem might be further aggravated by the alveolar increase of gas pressure due to mechanical ventilation. Conceptualizing lung capillary flow as taking place between parallel alveolar sheets (Sobin et al., 1970), their compression would also deform the capillary lumen from a circular to an ellipsoidal shape, further restricting RBC passage and lung capillary blood flow.

Furthermore, capillaries are lined by endothelial cells, which operate like osmometers (Mazzoni et al., 1990) that regulate their volume in order to balance hydraulic and osmotic pressures acting on the blood-containing capillary walls. In this system, the hydraulic pressure to which they are subjected is generated by the heart, minus the pressure losses due to viscous and dynamic pressure effects, and the outside pressure imparted by the atmosphere. This additional intra-capillary effect lowers capillary wall hydrostatic pressure favoring capillary edema further decreasing capillary blood flow. In this context, the decrease in diameter in pipe flow is partly corrected by the increase of flow velocity, which leads to a decrease of blood’s lung residence time necessary for re-oxygenation (Kreimeier and Messmer, 2002). This phenomenon may be further aggravated by the concomitant increase in vessel wall shear stress, increasing nitric oxide production (Østergaard, 2021), inducing arteriolar and venular vasodilatation, further increasing blood flow velocity, thus additionally lowering blood oxygenation.

The lowering of intracapillary hydrostatic pressure during mechanical ventilation might induce deleterious effects of endothelial edema. These effects are not restricted to hindering RBC passage in pulmonary capillaries and extend to all blood cellular components with RBC dimensions and greater. For example, neutrophils, whose diameter is greater than RBC’s, makes pulmonary capillaries to be particularly susceptible to the effects of increased mechanical hindrance due to endothelial edema (Park et al., 2019), an effect that the authors observed directly in an experimental animal model using a custom-made video laser scanning confocal microscope. A model of white blood cell passage through the pulmonary microcirculation leads to the same conclusion (Dupire et al., 2020).

The decrease in capillary diameter due to capillary compression and endothelial swelling tends to reduce FCD. Notably direct *in vivo* studies of the microcirculation in awake mammals revealed that the maintenance of FCD is critical to survival in circulatory shock. The decrease in this parameter to values below 45% of the normal indicates conditions leading to tissue/organ demise (Perl et al., 1975).

The reduction in FCD stems primarily from the decrease in capillary lumen diameter due to endothelial edema associated with the drop in hydraulic capillary pressure, as previously indicated. Capillaries can be conceptualized as tunnels in a gel matrix (Østergaard, 2021) whose internal diameter depends, in part, on the hydration of the matrix in which they are embedded, a parameter of limited variability. However capillary tunnels are lined by thin, flat, pancake-like osmometric endothelial cells. The latter behave like osmometers, whose volume and intrusion into the capillary lumen are regulated by the interaction of local hydraulic and osmotic plasma pressures (Smith et al., 1985; Mazzoni et al., 1988).

In hemorrhagic shock, the change in osmotic balance increases endothelial cell volume, which lowers capillary diameter and decreases hydraulic capillary pressure, ultimately, reducing FCD (Mazzoni et al., 1989). This physiological cascade is due to endothelial cell edema causing capillary constriction that traps RBCs, interrupting capillary blood flow. Intravenous introduction of hypertonic saline reverses this process, increasing capillary lumen, flow, and FCD in about 30 min (Smith et al., 1985), approximately the same time frame predicted by our modeling of fluid extraction from inundated alveoli treated with hypertonic NaCl infusions.

We assumed the fluid in a partially inundated alveolus to be isosmotic with circulating plasma. The osmotic tissue barrier separating the two is partially effective, as evidenced by the lung not being fully filled with fluid. The intravenous increase in plasma osmolarity increases osmotic flow from the alveolus into the capillaries, “drying” portions of the lung. This process continues until diffusion of the osmotic agent from the bloodstream into the alveolus equilibrate concentrations, allowing the return of plasma fluid into the alveolus. However, this is a much slower process than fluid extraction since it is driven by the changes in osmotic pressure due to diffusion.

An important consideration, left for a follow-up study, is whether and how hyperosmolar and possibly hyperoncotic conditions affect the endothelial barrier function, including the maintenance of endothelial cell function and glycocalyx properties (Aldecoa et al., 2020). Experimental evidence reveals that infusion of hyperosmolar sucrose in lung venular capillaries enhances the capillary barrier function, as quantified by the capillary hydraulic conductivity; this finding suggests that hyperosmolar therapy might be beneficial in lung inflammatory disease (Safdar et al., 2003). The combined genetic- and protein-level analysis of Kang et al. (2023) leads to a similar conclusion. This study demonstrated that microcirculatory hyperosmolarity upregulates cell-cell junction tension, showing that intravascular hyperosmotic conditions mechanically stabilize the vascular barrier, thus providing an additional therapeutic effect.

## 5 Conclusion

Experimental evidence suggests that, when used in ARDS treatment, hypertonic infusions rapidly (in tens of minutes) restore microvascular flow, normalizing capillary dimension, flow, and function (Mazzoni et al., 1990). Our mathematical model provides a mechanistic explanation for this effect. That is in contrast to mechanical ventilation, which might require up to a month to clear ARDS-affected lungs (Nolley et al., 2023). The results of this study highlight the extraordinary speed with which the effects of the intravenous infusion of hypertonic NaCl solution take place. This treatment strategy is cost-effective and has limited side effects. We posit that our results, and available experimental and clinical data, support consideration of this approach for aiding the treatment of lung edema, by facilitating oxygen diffusion into capillaries.

Supporting our recommendation, we note that hypertonic resuscitation is a procedure with little adverse side effects. Furthermore, the use of hypertonic hyperosmotic resuscitation to treat hypovolemia and heart function in hypovolemic shock was subjected to clinical studies, demonstrating that outcomes of this treatment are equivalent to blood transfusion since it increases oxygen delivery (Kreimeier and Messmer, 2002), leading to its use in some European ambulance services.

## Data Availability

The original contributions presented in the study are included in the article/supplementary material, further inquiries can be directed to the corresponding author.
